# Effect of different recovery modes during resistance training with blood flow restriction on hormonal levels and performance in young men: a randomized controlled trial

**DOI:** 10.1186/s13102-022-00442-0

**Published:** 2022-03-25

**Authors:** Vahid Fekri-Kourabbaslou, Sara Shams, Sadegh Amani-Shalamzari

**Affiliations:** Department of Exercise Physiology, Faculty of Physical Education and Sports Sciences, Kharazmi University, Tehran, Iran

**Keywords:** Blood flow occlusion, Active recovery, Hormone, Anaerobic performance, Muscle endurance

## Abstract

**Background:**

Resistance training with blood flow restriction (BFR) results in hypertrophy, and its magnitude depends on various training variables. This study aimed to compare the long-term effect of passive recovery (PR) and active recovery (AR) during low-intensity resistance training with BFR on hormonal levels and performance in young men.

**Methods:**

In the randomized clinical trial, 20 men were randomly divided into PR and AR groups during resistance training with BFR. The intervention consisted of six upper and lower body movements with 30% of one maximum repetition (1RM), three sessions per week for six weeks. Both groups wore pneumatic cuffs on the proximal part of thighs and arms. The cuff pressure was 60% of the calculated arterial blood occlusion and increased 10% every two weeks. The AR group performed seven repetitions in 30 s break between sets by one second for concentric and eccentric phases and two seconds rest, and the other group had passive rest. The blood samples and a series of performance tests were gathered before and after the intervention. A repeated measure ANOVA was used to analyze data.

**Results:**

AR and PR interventions significantly improved the C-reactive protein (CRP) (− 38% vs. − 40%), Lactate dehydrogenase (LDH) (− 11% vs. − 3%), Sargent jump (9% vs. 10%), peak power (20% vs.18%), and average power (14% vs. 14%), upper 1RM (8% vs. 8%) and no significant differences were observed between groups. The AR intervention significantly increased growth hormone (GH) (423% vs. 151%, *p* = 0.03), lower body 1RM (18% vs. 11%) and muscle endurance (34% vs. 22% for the upper body, *p* = 0.02 and 32% vs. 24% for the lower body, *p* = 0.04) than the PR group. The PR intervention further increased the minimum power than the AR group (19% vs. 10%). There were no significant changes in testosterone (*p* = 0.79) and cortisol (*p* = 0.34) following interventions.

**Conclusion:**

The findings indicated that by increasing muscle activation and higher metabolic load, AR during resistance training with BFR might cause more remarkable improvements in serum GH, muscle strength, and endurance. Thus, to gain further benefits, AR during training with BFR is recommended.

*Trial registration*: IRCT20191207045644N1. Registration date: 14/03/2020. URL: https://www.irct.ir/search/result?query=IRCT20191207045644N1

**Supplementary Information:**

The online version contains supplementary material available at 10.1186/s13102-022-00442-0.

## Introduction

Resistance training is recommended to maintain and promote muscle mass and strength; hence, it has health benefits [1], but some individuals are reluctant or unable to lift heavyweights. In recent decades, training with blood flow restriction (BFR) has become popular as an alternative to traditional resistance training in training settings. In this way, a pneumatic cuff or elastic band is used to reduce blood flow and occlude venous return that induces an ischemic state in muscle tissue[2]. Resistance training has a high mechanical, low metabolic load [3]; however, during BFR, the metabolic load increases and elicits the same adaptations similar to heavy training [4]. Therefore, low-load resistance training with BFR is recommended to increase muscle mass.

Studies have shown that resistance training with BFR induces hypertrophy and increases muscle size and strength [5–7]. Some mechanisms have been proposed for these adaptations, including an increase in the recruitment of fast-twitch fibers (type II) [2], muscle cell swelling [8], production of reactive oxygen species like nitric oxide, activation of anabolic pathways [9], and increased secretion of catecholamine and anabolic hormones such as growth hormone (GH) and testosterone due to anaerobic metabolism and lactate accumulation [10–13]. Increased growth hormone and testosterone were reported following training with BFR [14]; these anabolic hormones promote muscle growth and muscle strength [15], which subsequently enhance muscle endurance. Therefore, BFR training could elicit specific adaptations in active muscles.

Training adaptation depends on the exercise variables. In training with BFR, several factors, including the occlusion pressure, the type of occlusion, and the occlusion duration, could affect the adaptations [16]. In resistance training, a combination of variables such as type of muscle contraction (concentric and eccentric), training volume (number of sets and repetitions), the intensity of exercise, involved muscle groups, and also the recovery between sets induce different physiological adaptations [17, 18]. The type of recovery between resistance exercises is an influential factor in muscle adaptation. Using active recovery by depleting creatine phosphate reserves and increasing levels of intracellular glycolytic metabolites increases the activity of sympathetic nerves by stimulating chemical receptors and consequently increasing the resistance of peripheral arteries [10, 19, 20]. This mechanism increases heart rate and cardiac output, increases vasodilator substrates such as nitric oxide, and increases exercise load during subsequent movement by increasing the exercise duration [19, 21, 22]. In general, the more the involvement of the anaerobic system and metabolic stress, the more significant hormonal and muscular responses and adaptations.

Active recovery can significantly reduce oxygen saturation (SAO_2_) and anaerobic work capacity, an increase in total metabolic rate, and lactate production. In ischemic conditions or lack of oxygen in the muscle during BFR training, the downstream signaling has been activated and stimulates angiogenesis and adaptations [23–25]. However, no study has examined the effectiveness of AR during resistance training with BFR on physiological and performance responses and adaptations. Thus, we hypothesized that AR might have profound effects on hormonal and performance adaptations. Therefore, this study was designed to investigate the effects of AR and PR during resistance training with BFR on GH, cortisol, testosterone hormones, and performance indices.

## Materials and methods

### Study design

**Fig. 1:**
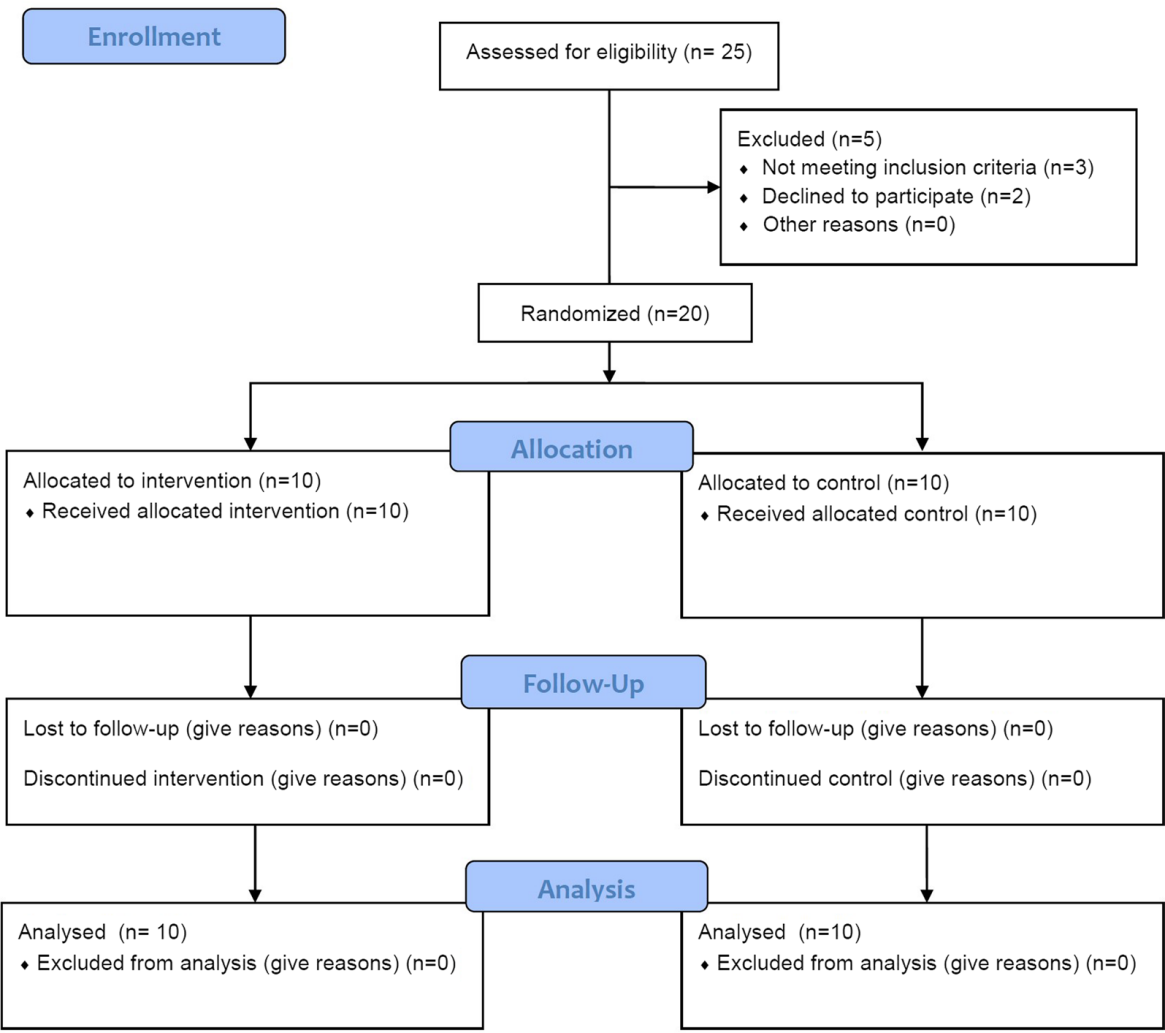
Schematic overview of study timeline (CONSORT flow diagram)

This randomized control trial, parallel groups, was conducted to investigate the effectiveness of active or passive recovery during resistance training with BFR on hormonal responses and adaptations and anaerobic capacity and explosive power. At the first visit, participants were familiarized with the training protocols; anthropometric characteristics were also measured. Next week, physiologic characteristics, anaerobic capacity, explosive power, one repetition maximal, muscle endurance were assessed interspersed after recovery in four sessions. A schematic overview of the study is presented in Fig. 1.

### Participants

Twenty young men volunteered to participate in this study. The anthropometric characteristics of participants are presented in Table 1. The inclusion criteria were physically active men aged 20–26 years, nonsmokers, BMI < 25 and without any chronic cardiovascular, metabolic, and neuromuscular disease, no use of supplements, and without any specific training routines. To estimate the number of participants in each group, a sample size calculation was performed using G*Power Software for repeated measured ANOVA, using a rejection criterion of 0.05 and 0.9 [1-beta] power, and medium effect (f = 0.4), a minimum of 10 participants need to each group. Exclusion criteria included the use of dietary supplements, any injury or discomfort from exercise, and absenteeism for more than two sessions. A third person who was not in the research group allocated eligible participants randomly into two groups; passive recovery during resistance training with BFR (PR; n = 10) and active recovery during resistance training with BFR (AR; n = 10). The risks and benefits of the study were explained to participants, and written informed consent was obtained from all subjects before initial assessments. All procedures performed in studies involving human participants were under the Helsinki statement regarding human research. The study was approved by the Ethic committees for the Sport Sciences Research Institute of Iran (approval number: IR.SSRI.REC.1398.129). In addition, this research was registered (14/03/2020) in the Iranian Registry of Clinical Trials (IRCT) with registration number: IRCT20191207045644N1.

**Table 1:** Anthropometric characteristics of participants

Group	Age (years)	Height (m)	Body mass (kg)		BMI (kg/m^2^)	
			Pre	Post	Pre	Post
PR	22.38 ± 2.32	1.77 ± 0.04	70.00 ± 5.61	71.05 ± 4.78	22.16 ± 1.36	22.48 ± 1.00
AR	22.38 ± 1.92	1.81 ± 0.04	72.60 ± 6.51	73.25 ± 5.99	21.85 ± 1.25	22.05 ± 1.18

BMI, body mass index; PR, passive recovery; AR, active recovery

### Test procedures

One week before and 48 h after the training period, participants attended the laboratory to complete physiological and performance tests. At first, body mass (digital weighing scales, Seca 769, Germany), height (stadiometer, Seca 213, Germany), body mass index(kg/m2), systolic and diastolic blood pressure (sphygmomanometer, Riester, Germany), and thigh and arm circumference (tape meter) were measured.

On the same day, the participants completed a 30 s anaerobic Wingate test on a stationary bicycle ergometer (Monark Model 894E, Monark, Vansbro, Sweden) to assess anaerobic capacity, including peak power (PP), minimum power (MP), and average power (AP). After completing the warm-up [5 min with 60 rpm], subjects were immediately given a 5 s countdown to the automatically controlled beginning of the test. During the trial, subjects pedaled as fast as they could for 30 s while remaining seated. The resistance load for the Wingate test was set equivalent to 7.5% of each subjects' body mass. Players were verbally encouraged during all tests to perform to their maximum ability.

In the next session, 48 h later, the participants performed the Sargent jump test based on the instruction in the previous study. Subjects stood on their feet on the ground, then reached up as high as possible with one hand closest to the wall and marked the wall with the tips of the fingers (M1). Later stood away from the wall and from a static position, jumped as high as possible, and marked the wall with fingers (M2). The researcher measured and recorded the distance between M1 and M2. The test was repeated three times, and the best of 3 attempts was recorded.

On the same day, for the 1RM assessment, participants choose a load that they can lift at most one repetition. After doing that, the researcher put the numbers of repetitions and the amounts of weight in the Berzycki formula to calculate the one-repetition maximum (1RM) for each exercise. in the following sessions on another day, all subjects were asked to perform each exercise with 40% of 1RM [2 s for each repetition with metronome]. When participants lagged twice from metronome rhythm, the activity was prevented. The tests were repeated 48 h after the training intervention period, respectively.

### Training protocol

The training protocol consisted of three sessions per week for six weeks [18 sessions], and all the movements for the groups were the same, including lat pulldown, chest press, shoulder press, hack squat, seated leg extension, and lying leg curl. The order of exercises alternated between upper body and lower body. The contraction tempo was 1 s for both concentric and eccentric contractions by 30% of 1RM. The training protocol for the PR (passive recovery) group consisted of 4 sets with 30–15–15–15 repetitions with 30 s rest between sets and 1 min rest between exercises. Still, participants in AR (active recovery) group have done three sets with 30–15–15 repetitions with seven repetitions in 30 s rest between sets and 1-min passive recovery between exercises. During AR, subjects performed seven repetitions in 30 s, 1 s for concentric, 1 s for eccentric phase, and 2 s rest, then continued this rhythm.

During training sessions, both groups wore right and left leg/arm pneumatic cuffs (5 cm width, 120 cm length for leg, and 60 cm for arms, Ghamatpooyan, Tehran, Iran) placed on the proximal part of thighs and arms. To determine the cuff pressure during the training sessions, the arterial blood flow occlusion was estimated using the following two formulas for the upper and lower body [26].

Lower body arterial occlusion (mmHg) = 5.893 × (Thigh circumference) + 0.734 × (diastolic blood pressure) + 0.912 × (systolic blood pressure) − 220.046.

Upper body arterial occlusion (mmHg) = 0.514 × (systolic blood pressure) + 0.339 × (diastolic blood pressure) + 1.461 × (Arm circumference) + 17.236.

The cuff pressure in training sessions was ~ 60% of the calculated arterial blood occlusion in the first two weeks, which increased to 10% every two weeks to reach 80% of the calculated arterial occlusion in weeks 5 and 6. The cuffs were inflated and maintained throughout all sets and repetitions and deflated in 1 min break period between stations. The rate of perceived exertion (RPE) was recorded as a mental indicator for monitoring training intensity.

### Hormonal analysis

A 5 ml blood sample was collected 48 h before and after training intervention following an overnight fast and immediately after the first and last training sessions from the antecubital vein. 2 ml of samples were collected in‌ ethylenediaminetetraacetic (EDTA) tubes for plasma lactate assessment, and 3 ml was obtained with separator gel vials no EDTA to the evaluation of serum GH, lactate dehydrogenase (LDH), C-reactive-protein (CRP), testosterone, cortisol. Blood samples were immediately centrifuged (4 °C, 3000 rpm) for 10 min after collection to isolate serum and were then stored at − 20° C. Serum samples were analyzed by enzyme immunoassay for free testosterone (testosterone kit IBL, RE52151, Hamburg, Germany), cortisol (cortisol kit IBL, RE52061, Hamburg, Germany), GH (growth hormone kit IBL, MG59121, Hamburg, Germany), and CRP (CRP kit IBL, EU59131, Hamburg, Germany). Plasma lactate and lactate dehydrogenase (LDH) activities were measured using colorimetry and spectrophotometry methods (Pars Azmoon Inc., Iran) by an auto-analyzer (Hitachi, USA).

### Statistical analysis

Data were analyzed using the statistical package of social sciences (SPSS, IBM, v19) and significance levels set at p ≤ 0.05. Data presented as mean (SD). An independent sample t-test was performed to analyze data in the baseline. The repeated measured ANOVA was performed to interpret the variables (Time, Group, and Time*Group). The assumptions of data normality (Shapiro–Wilk's test), and homogeneity of variance (box plot), were confirmed. For analysis of the acute response of GH, LDH, and lactate, we subtracted the acute response values from baseline in the first and last session and used these data for statistical analysis. Furthermore, the magnitude of differences in the dependent measures within groups was reported using the effect size (ES). Effect size was calculated by the change score divided by the SD of the change score to remove the influence of the sample size [27]. The ES statistic was interpreted using the following criteria: trivial (< 0.20), small (0.20–0.49), moderate (0.50–0.79), and large effects (> 0.80).

## Results

The outputs of the independent sample t-test demonstrated that there were no differences between groups in the baseline in all variables (p > 0.05).

### Anthropometric variables

Table 2 shows the measured anthropometric, physiological, and performance variables and magnitude of change pre- to post-training for two groups. We found a significant main time effect for body mass (F_1,9_ = 7.74 *p* = 0.021, *ηp*^*2*^:0.46), through no significant group (F_1,9_ = 0.54 *p* = 0.482, *ηp*^*2*^:0.057) and interaction effect (F_1,9_ = 0.35 *p* = 0.568, *ηp*^*2*^:0.04) was observed. In addition, for BMI, there was a significant main time effect (F_1,9_ = 6.56 *p* = 0.031, *ηp*^*2*^:0.42), through no significant group (F_1,9_ = 0.36 *p* = 0.562, *ηp*^*2*^:0.04) and interaction effect (F_1,9_ = 0.30 *p* = 0.593, *ηp*^*2*^:0.03) was observed.

**Table 2:** Performance and blood markers before and after the interventions

Variables	Groups	Pre	Post	% change	Cohen,s d	P time* group interaction
Body mass(kg)	PR	70.00 (5.61)	71.05 (4.78)	1.62	0.55	0.568
	AR	72.60 (6.51)	73.25 (5.99)	0.95	0.86	
BMI(kg/m^2^)	PR	22.16 (1.36)	22.48 (1.00)	1.59	0.50	0.593
	AR	21.85 (1.25)	22.05 (1.18)	0.94	0.82	
Upper one repetition maximum (kg)	PR	64.75 (10.85)	69.88 (11.42)	7.99	2.80	0.844
	AR	68.63 (7.98)	73.88 (7.83)	7.79	4.51	
Lower one repetition maximum (kg)	PR	105.00 (16.03)	115.87 (16.01)	10.66	2.82	0.003*
	AR	100.00 (19.27)	117.13 (18.57)	18.15	4.21	
GH (ng/ml)	PR	0.24 (0.21)	0.49 (0.29)	150.90	1.45	0.030*
	AR	0.22 (0.15)	0.88 (0.48)	423.35	1.57	
LDH (U/L)	PR	291.10 (55.45)	278.20 (33.32)	− 2.57	− 0.36	0.189
	AR	285.50 (25.27)	253.50 (23.85)	− 11.12	− 2.10	
CRP (mg/L)	PR	2.87 (0.30)	1.65 (0.74)	− 39.79	− 1.21	0.730
	AR	2.71 (0.41)	1.66 (0.86)	− 38.35	− 1.24	
Testosterone (ng/ml)	PR	5.07 (1.34)	5.40 (1.02)	12.07	0.24	0.787
	AR	6.16 (1.24)	6.27 (1.19)	4.47	0.07	
Cortisol (ng/ml)	PR	8.96 (2.08)	7.70 (1.85)	− 12.01	− 0.66	0.340
	AR	7.92 (2.78)	7.47 (2.05)	− 2.14	− 0.12	
Sargent jump (cm)	PR	48.50 (4.08)	53.20 (4.34)	9.81	2.03	0.872
	AR	52.20 (4.15)	57.00 (3.94)	9.32	2.96	
Peak power (W)	PR	570.53 (54.58)	668.36 (61.06)	17.45	2.23	0.559
	AR	596.14 (72.69)	709.66 (70.26)	19.73	2.56	
Average Power (W)	PR	424.45 (37.06)	480.85 (34.42)	13.53	2.93	0.956
	AR	435.03 (56.54)	492.17 (48.47)	14.08	1.55	
Minimum power (W)	PR	251.42 (35.51)	297.62 (45.69)	18.69	1.58	0.049*
	AR	252.73 (40.37)	274.68 (44.38)	9.61	0.63	
Power drop (%)	PR	55.69 (6.48)	54.97 (8.51)	− 0.69	− 0.09	0.189
	AR	57.64 (3.84)	61.23 (5.06)	6.41	0.77	
Upper body muscle endurance (REP)	PR	30.80 (3.11)	37.70 (3.30)	22.43	2.16	0.022*
	AR	31.60 (4.64)	42.20 (6.42)	33.53	2.93	
Lower body muscle endurance (REP)	PR	32.80 (4.44)	40.60 (3.95)	23.67	1.92	0.035*
	AR	30.90 (4.17)	40.80 (4.70)	32.13	2.22	

GH, growth hormone; LDH, lactate dehydrogenase; CRP, c-reactive protein; PR, passive recovery; AR, active recovery; REP, repetition W: wat; * Significant difference between groups

### Blood variables

**Fig. 2:**
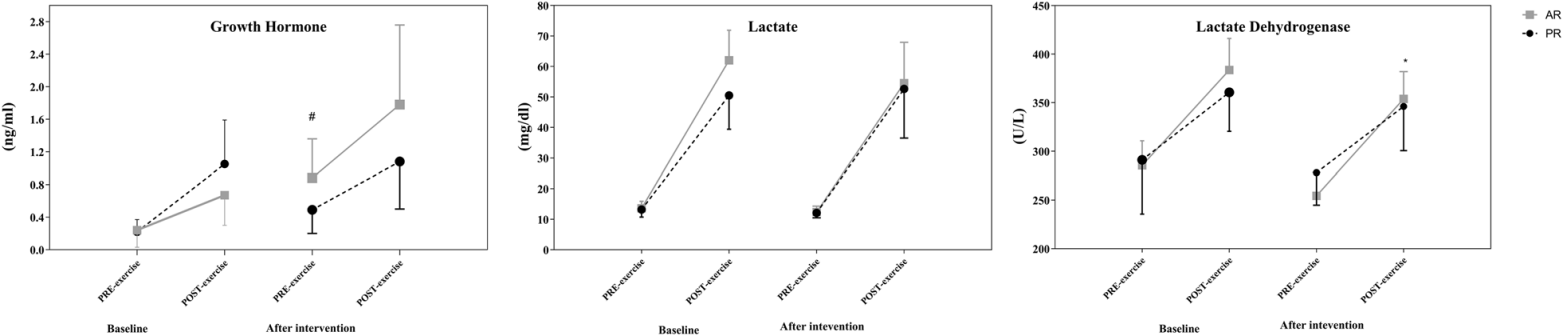
Acute response of growth hormone, lactate, and lactate dehydrogenase before and after the intervention. *significant difference in the response of variables between groups, #significant difference in the resting level between groups

The results of repeated measures ANOVA demonstrated there were significant main time (F_1,9_ = 51.7 *p* = 0.001, *ηp*^*2*^:0.85), and interaction time × group (F_1,9_ = 6.6 *p* = 0.030, *ηp*^*2*^:0.42) for GH levels; however, the main group effect (F_1,9_ = 1.60 *p* = 0.236, *ηp*^*2*^:0.15) was not significant. The rest level of GH increased significantly in the AR group. We measured the acute response of GH to the first and last training sessions, and the data is shown in Fig. 2. There was no significant main group (F_1,9_ = 2.92 *p* = 0.121, *ηp*^*2*^:0.25), time (F_1,9_ = 2.36 *p* = 0.159, *ηp*^*2*^:0.21) and interaction (F_1,9_ = 0.19 *p* = 0.667, *ηp*^*2*^:0.01) effect for GH response after intervention. For LDH, a significant main time effect (F_1,9_ = 18.14 *p* = 0.002, *ηp*^*2*^:0.67), and no significant group (F_1,9_ = 1.28 *p* = 0.286, *ηp*^*2*^:0.13) and interaction effect (F_1,9_ = 2.02 *p* = 0.189, *ηp*^*2*^:0.18) was perceived. There was a significant main group (F_1,9_ = 8.16 *p* = 0.019, *ηp*^*2*^:0.48), through no significant difference time (F_1,9_ = 0.01 *p* = 0.965, *ηp*^*2*^:0.01) and interaction (F_1,9_ = 0.07 *p* = 0.796, *ηp*^*2*^:0.01) effect was observed for LDH response to first and last training sessions. The response of LDH to exercise increased further in the AR group after the intervention. In addition, there was no significant main group (F_1,9_ = 3.40 *p* = 0.098, *ηp*^*2*^:0.27), time (F_1,9_ = 0.26 *p* = 0.624, *ηp*^*2*^:0.03), and interaction (F_1,9_ = 0.1.57 *p* = 0.242, *ηp*^*2*^:0.15) effect for the response of lactate to first and last training sessions (Fig. 2).

For testosterone, we observed no significant main time (F_1,9_ = 1.14 *p* = 0.314, *ηp*^*2*^:0.11), group (F_1,9_ = 3.44 *p* = 0.097, *ηp*^*2*^:0.28), and interaction effect (F_1.9_ = 0.07 *p* = 0.787, *ηp*^*2*^:0.01). Moreover, there was no significant main time (F_1,9_ = 3.71 *p* = 0.086, *ηp*^*2*^:0.29), group (F_1,9_ = 0.517 *p* = 0.490, *ηp*^*2*^:0.054) and interaction effect (F_1,9_ = 1.01 *p* = 0.340, *ηp*^*2*^:0.10) for cortisol. In addition, the intervention led to a significant main time effect (F_1,9_ = 45.65 *p* = 0.001, *ηp*^*2*^:0.84), through no significant group (F_1,9_ = 0.09 *p* = 0.760, *ηp*^*2*^:0.01) and interaction effect (F_1,9_ = 0.127 *p* = 0.730, *ηp*^*2*^:0.014) was observed for CRP level. The serum CRP concentration was decreased in both groups (Table 2).

### Performance variables

There was a significant main time (F_1,9_ = 137.28 *p* = 0.001, *ηp*^*2*^:0.95), but no main group (F_1,9_ = 1.04 *p* = 0.341, *ηp*^*2*^:0.13) and interaction effect (F_1,9_ = 0.04 *p* = 0.844, *ηp*^*2*^:0.01) for upper 1RM. For the lower 1RM, the intervention led to a significant main time (F_1,9_ = 138.93 *p* = 0.001, *ηp*^*2*^:0.95), and interaction effect (F_1,9_ = 18.93 *p* = 0.003, *ηp*^*2*^:0.73), but with no main group effect (F_1,9_ = 0.04 *p* = 0.836, *ηp*^*2*^:0.01). The change percentage in the AR group (18.2%) was higher than the PR group (10.7%) in the lower 1RM.

The intervention resulted in a significant main time (F_1,9_ = 15.78 *p* = 0.003, *ηp*^*2*^:0.64), and interaction effect (F_1,9_ = 5.15 *p* = 0.049, *ηp*^*2*^:0.36), but no main group effect (F_1,9_ = 0.25 *p* = 0.625, *ηp*^*2*^:0.02) for minimum power levels. The change percentage in the PR group (18.3%) was higher than the AR group (8.6%) in the minimum power. However, the main time effect were observed for peak power (F_1,9_ = 425.66 *p* = 0.001, *ηp*^*2*^:0.98), and average power (F_1,9_ = 76.27 *p* = 0.001, *ηp*^*2*^:0.89), through no significant group (peak power:F_1,9_ = 2.93 *p* = 0.121, *ηp*^*2*^:0.25; average power: F_1,9_ = 0.45 *p* = 0.520, *ηp*^*2*^:0.05) and interaction effect (peak power:F_1,9_ = 0.37 *p* = 0.559, *ηp*^*2*^:0.04; average power: F_1,9_ = 0.003 *p* = 0.956 *ηp*^*2*^:0.001) were observed.

In addition, a significant main time effect (F_1,9_ = 73.50 *p* = 0.001, *ηp*^*2*^:0.89), and no significant group (F_1,9_ = 3.10 *p* = 0.112, *ηp*^*2*^:0.26) and interaction effect (F_1,9_ = 0.03 *p* = 0.872, *ηp*^*2*^:0.003) was perceived for Sargent jump. Furthermore, for muscle endurance, the intervention resulted in a significant time (upper body: F_1,9_ = 105.01 *p* = 0.001, *ηp*^*2*^:0.92; lower body: F_1,9_ = 132.93 *p* = 0.001, *ηp*^*2*^:0.94) and interaction (upper body: F_1,9_ = 7.70 *p* = 0.022, *ηp*^*2*^:0.46; lower body: F_1,9_ = 6.11 *p* = 0.035, *ηp*^*2*^:0.41), through there was no significant main group effect (upper body:F_1,9_ = 2.34 *p* = 0.16, *ηp*^*2*^:0.21; lower body: F_1,9_ = 0.85 *p* = 0.37, *ηp*^*2*^:0.087). The increase rate in the AR group was more than the PR group in upper and lower body muscle endurance. At last, the result of repeated measures ANOVA showed there was no significant main group (F_1,9_ = 1.93 *p* = 0.198, *ηp*^*2*^:0.18), time (F_1,9_ = 1.11 *p* = 0.319, *ηp*^*2*^:0.11), and interaction effect (F_1,9_ = 2.02 *p* = 0.189, *ηp*^*2*^:0.19) for power drop.

## Discussion

This study aimed to determine the effectiveness of active and passive rest between repetitions of a period of resistance training with BFR on hormonal levels and performance of young men. The findings show that AR during resistance training with BFR results in further increments in GH levels, lower 1RM, and muscle endurance; PR during resistance training with BFR improved minimum power. Moreover, inflammatory markers, the CRP and LDH, decreased in both groups; anthropometric variables and some performance indices such as anaerobic power and Sargent test increased in both groups. However, both interventions had no significant effect on testosterone and cortisol levels. Therefore, since the AR intervention led to further increased muscle endurance and lower body strength and GH secretion, we recommend this intervention to obtain more benefits during resistance training with BFR.

To our knowledge, no studies are investigating the effect of resistance training and blood flow restriction with active recovery on a hormonal level and performance, and the research literature in this area is limited. Brito et al. (2011) investigated the effect of resistance training with active rest on reducing exercise-induced pressure in hypertensive older women and concluded that it causes more significant exercise-induced hypotension than the passive rest group [19]. The more exercises involve the anaerobic system, the greater stimulation of GH secretion [10, 28]. In other words, BFR reduces oxygen and ultimately leads to increased lactate production [10, 12]. Evidence suggests that low pH stimulates sympathetic neuronal activity through chemical reflexes, intramuscular mechanical receptors, and group III and IV afferent fibers. Recently, similar pathways have been shown to play an essential role in regulating the pituitary secretion of GH. Accumulated intracellular metabolites may also stimulate GH changes through group III and IV afferents, which are sensitive to adenosine, K^+^, H^+^, hypoxia, and AMP changes. It can be deducted that the further increase in these metabolites during BFR exercise with active rest causes higher stressful reflexes such as increased heart rate and blood pressure and production of more glycolytic metabolites (e.g., lactate), thus facilitating an increase in GH secretion [22, 29].

CRP is a sensitive inflammatory marker produced by hepatocytes in response to inflammatory markers like interleukin-6 (IL-6), IL-1, and tumor necrosis factor-alpha (TNF-α) [30]. Studies have shown that physical activity reduces inflammation by improving endothelial function and that exercise decreases CRP production by reducing or inhibiting cytokines [31, 32]. It has been shown that regular physical activity can reduce circulating levels of inflammatory markers [32, 33]. Many researchers have suggested that a decrease in CRP levels is independent of weight loss and is due to training only with increased fitness and physiological effects due to its anti-inflammatory activity [34]. Research has shown that intense exercise increases the levels of heat shock proteins 72 and increases these proteins play an essential role in cell protective pathways and reduce ischemic injury [35]. In this regard, researchers investigated the effect of resistance training with BFR on CRP in healthy individuals [36, 37]. The results showed that resistance training with BFR did not significantly affect CRP levels in healthy individuals [36, 37]. It is perceived that their baseline values ​​were less than enough to make a significant difference between the groups. Also, Madarameh et al. (2013), in an experimental study, examined inflammatory and hemostatic responses to resistance training with BFR in patients with ischemic heart disease, and the results showed that intervention did not affect inflammatory and hemostatic responses [38]. In the present study, we tried to control gender, smoking, duration, and exercise intensity.

The magnitude of changes in muscle damage induced by resistance training can be influenced by the individual’s training status, exercised muscle group, adopted intensity, repetition volume [muscle failure vs. not failing], and execution pace. Therefore, the contradictory results about the effects of resistance training with BFR on muscle damage can be justified by using different protocols and samples investigated in studies [39]. In this regard, studies by Neto et al. (2018) which examined the acute effect of resistance training with BFR, showed no significant change in serum lactate dehydrogenase levels [40]. On the other hand, Clarkson and Thompson also stated that regular physical activity decreases muscle damage indicators, including LDH [41]. In the present study, LDH levels decreased in all groups after six weeks, but no significant difference was observed. Moreover, six weeks of training lead to adaptation, not muscle damage.

Testosterone and cortisol are the most critical anabolic and catabolic steroid hormones. Factors affecting the response of testosterone to exercise include intensity, volume, duration, rest, active muscle groups, history, and experience of resistance training [42]. A study has reported that resistance training and high-intensity exercises increase total testosterone concentration in men [43]. The possible mechanism behind the acute increase in testosterone with low-intensity training and BFR with active recovery may be due to increased lactate and concentration of catecholamines, which usually increase during training that stimulates testicular Leydig cells to increase testosterone [44–46]. However, the possible explanation for why BFR training does not increase testosterone levels at rest is likely due to the low training intensity.

On the other hand, cortisol is a glucocorticoid secreted by the adrenal cortex and changes in stressful conditions such as environmental effects, emotional pressure, and exercise. The results of a study by Fujita et al. on young men showed that cortisol levels were higher in the exercise group with occlusion than in the non-restricted group [47]. Also, the researchers believe that although the intensity of BFR training is lower than traditional resistance training, closing the cuff and creating a hypoxic environment, also active recovery locally increase lactate production and stimulate the hypothalamic-pituitary axis that increases the secretion of cortisol as a hormonal stress factor by increasing physiological pressure [48–50]. In the present study, changes in testosterone and cortisol levels before and after the test and between groups were not significant, which could be because of measuring time of hormones level. These hormone levels increase immediately after or during exercise [51, 52] and decrease after training.

Research shows that resistance training positively affects anaerobic power and neuromuscular properties. Long-term resistance training can improve anaerobic power by altering the nervous and muscular systems, increasing anaerobic enzymes, energy production, intracellular glycogen, or changing the type of muscle fibers [53]. Also, resistance training with blood restriction can cause more type II muscle fiber recruitment by accumulating metabolites such as lactate and reducing muscular PH, which is one of the main adaptation mechanisms related to this method [10–13, 16, 54]. On the other hand, the lower the rest time, the greater in load and response of this exercise [19, 22]. The results indicate a significant and positive relationship between the power measured in the Wingate test, the isometric strength of the leg muscles, and the explosive strength of the leg muscles [55]. Therefore, the power measured in the Wingate test should also be increased due to an increase in the strength and explosive power of the leg muscles; in this regard, Cook et al. (2014) investigated the effect of resistance training with and without BFR on strength and power [56]. They concluded that low-load strength training with vascular occlusion has similar effects to traditional heavy-load resistance training on strength and anaerobic power, consistent with our study. The increase in strength observed in the present study (1RM) accompanied increased muscle endurance. Increased anabolic hormones, especially GH in the AR intervention, could be one of the causes of increased muscle endurance. In addition, recruitment of type 2 fibers [57] increases muscle glycogen storage [58] could be involved in promoting muscle endurance.

## Limitations

We confirm that there were some limitations in the present study. Firstly, the sample size was small; however, we used the effect size for pair data to monitor the influence of the sample size. Secondly, we could not control participants' sleeping time and diet during the intervention and test time, but we asked them not to change their habits. Finally, we did not measure body composition that helps us to interpret data; thus, we suggest researcher assess body composition by DEXA to determine the specific effects of AR and PR during training with BFR on the muscle mass of the extremities.

## Conclusion

The findings demonstrated that higher metabolic pressure in the AR intervention led to further increased muscle endurance and lower strength and GH secretion. Therefore, active recovery is recommended to gain more benefits during resistance training with BFR.

## Supplementary Information

**Additional file 1.** Row Data.

## Data Availability

The datasets generated and/or analyzed during the current study are available in a Additional file 1.

## Abbreviations

BFR: Blood flow restriction
PR: Passive recovery
AR: Active recovery
GH: Growth hormone
LDH: Lactate dehydrogenase
CRP: C-reactive protein
BMI: Body mass index
1RM: One maximum repetition
SAO2: Oxygen saturation
PP: Peak power
MP: Minimum power
AP: Average power
EDTA: Ethylenediaminetetraacetic
ES: Effect size
TNF-α: Tumor necrosis actor-alpha
IL-6: Interleukin-6
SPSS: Statistical package of social sciences
