# Correction: Effect of different recovery modes during resistance training with blood flow restriction on hormonal levels and performance in young men: a randomized controlled trial

**DOI:** 10.1186/s13102-022-00605-z

**Published:** 2022-12-13

**Authors:** Vahid Fekri-Kourabbaslou, Sara Shams, Sadegh Amani-Shalamzari

**Affiliations:** Department of Exercise Physiology, Faculty of Physical Education and Sports Sciences, Kharazmi University, Tehran, Iran

**Correction: BMC Sports Science, Medicine and Rehabilitation (2022) 14:47** 10.1186/s13102-022-00442-0

Following publication of the original article [1], the authors identified an error in the family name of Vahid Fekri-Kourabbaslou.

The incorrect author name is: Vahid Fekri-Kurabbaslou

The correct author name is: Vahid Fekri-Kourabbaslou

The author group has been updated above and the original article [1] has been corrected.
