# Supplementary material for: Effect of different recovery modes during resistance training with blood flow restriction on hormonal levels and performance in young men: a randomized controlled trial
Source: BMC Sports Sci Med Rehabil. 2022 Mar 25;14:47. doi: 10.1186/s13102-022-00442-0 (PMC8957130; doi:10.1186/s13102-022-00442-0)
Supplement: Supplementary file 1 — Additional file 1. Row Data. [file 13102_2022_442_MOESM1_ESM.pdf]

group	CRP1	CRP2	TESTESTRC	TESTESTRC	WEIGHT1	WEIGHT2	BMI1	BMI2
	2.4	3.3	6.17	7.5	73	73	23.3	23.3
	3.3	0.9	4.36	5.01	62	68	20.2	22.2
PR	2.9	1.9	4.51	3.74	81	80	25	24.6
	2.8	1.2	2.98	4.56	70	71.5	20.6	21.1
	2.5	2.2	5.66	6.33	65	65	21.7	21.7
	3.3	1.4	7.6	4.78	76	77	22.6	22.9
	3.1	0.6	5.84	5.76	68	69	22.4	22.7
	2.6	1.7	3.46	5.59	65	65	21.4	21.4
	2.9	1.7	5.07	5.41	70	71	22.2	22.5
	2.9	1.7	5.07	5.41	70	71	22.2	22.5
AR	2.1	1.3	4.66	5.64	67	68	20	20.3
	2.2	2.5	6.51	9.09	72	72	22.4	22.4
	2.9	0.2	5.26	5.3	66	67	20.3	20.6
	3.4	3	8.89	6.96	66	67	20.3	20.6
	2.4	0.5	7.21	5.91	88	87	23.6	23.3
	3.2	1.6	4.78	6.84	71	72	22.6	22.9
	2.9	2.4	5.63	5.7	76	77.5	23.1	23.7
	2.6	1.8	6.36	4.78	76	76	22.6	22.6
	2.7	1.66	6.16	6.27	72	73	21.8	22.05
	2.7	1.66	6.16	6.27	72	73	21.8	22.05

group	CORTISOL1	CORTISOL2	SARJENT1	SARJENT2	LACTATE1	LACTATE2
	8.72	7.41	43	49	12	14
	14.24	10.97	51	61	16	13
PR	10.11	8.79	44	48	16	11
	8.34	5.39	51	55	13	12
	7.8	8.07	50	53	11	10
	6.39	9.41	52	57	9	12
	8.21	7.14	43	47	12	10
	7.94	4.49	55	56	17	15
	8.96	7.71	48	53	13	12
	8.96	7.71	48	53	13	12
AR	15.25	11.95	56	63	14	14
	6.69	5.8	45	52	15	14
	7.48	5.84	54	60	13	16
	5.5	7.46	59	62	12	10
	3.14	6.89	55	57	10	10
	7.66	5.42	47	51	14	11
	7.45	6.37	50	54	19	13
	8.65	10.03	52	57	13	11
	7.72	7.47	52	57	13	12.38
	7.72	7.47	52	57	13	12.38

group	GH1	GH2	drop powe	drop powe	Peak Powe	Peak Powe	Minimum l	Minimum F
	0.1	0.3	55.41	46.88	632.66	676.97	282.06	359.58
	0.1	0.5	58.5	67.94	484.26	655.24	200.96	210.1
PR	0.1	0.15	64.51	59.59	644.23	744.16	228.6	300.67
	0.2	0.7	58.51	60.87	602.36	717.84	249.87	280.88
	0.15	0.6	55.01	59.37	562.67	650.78	253.13	264.39
	0.73	1.1	39.05	46.48	539.24	693.16	328.62	370.94
	0.05	0.07	56.22	39.14	489.67	514.91	214.35	313.35
	0.5	0.55	58.32	59.49	609.16	693.83	253.84	281.05
	0.24	0.49	55.69	54.97	570.53	668.36	251.43	297.62
	0.24	0.49	55.69	54.97	570.53	668.36	251.43	297.62
AR	0.3	1.9	63.2	70.06	632.45	734.03	232.68	219.76
	0.1	0.3	60.89	58.16	669.96	710.98	261.96	297.44
	0.07	1.15	50.71	53.52	622.76	791.45	306.95	367.82
	0.36	0.9	57.35	55.18	536.29	605.65	228.72	271.44
	0.55	1.25	53.12	66.92	642.38	753.74	301.11	249.33
	0.05	0.25	62.23	64.82	545.51	632.47	206.03	222.49
	0.25	0.7	57.23	59.76	439.14	629.64	187.78	253.39
	0.1	0.6	56.42	61.46	680.68	819.35	296.64	315.79
	0.22	0.88	57.64	61.23	596.14	709.66	252.73	274.68
	0.22	0.88	57.64	61.23	596.14	709.66	252.73	274.68

Power 2

group	Mean Pow	MeanPowe	LDH1	LDH2
	454.89	501.7	357	309
	382.92	456.79	296	267
PR	487.35	522.41	271	249
	426.56	499.36	260	258
	416.06	457.58	235	233
	443.85	532.05	393	353
	352.02	418.12	292	282
	432	458.79	305	275
	424.45	480.85	301	278
	424.45	480.85	201	278
AR	462.95	463.34	264	263
	483.89	520.48	309	286
	481.16	579.63	328	276
	405.18	438.54	260	217
	471.73	524.05	287	234
	374.6	427.48	314	283
	313.23	448.39	274	245
	487.5	535.46	249	225
	435.03	492.17	285	253
	435.03	492.17	285	253
